# Enhanced conductive body heat loss during sleep increases slow-wave sleep and calms the heart

**DOI:** 10.1038/s41598-024-53839-x

**Published:** 2024-02-26

**Authors:** Sebastian Herberger, Thomas Penzel, Ingo Fietze, Martin Glos, Alessandro Cicolin, Elisa Fattori, Daniela Grimaldi, Kathryn Reid, Phyllis Zee, Matteo Mason, Kurt Kräuchi

**Affiliations:** 1 Interdisciplinary Center of Sleep Medicine, Charité - Universitätsmedizin Berlin, Berlin, Germany; 2 Sleep Disorder Center, Department of Neurosciences, University of Torino, AOU Città della Salute e della Scienza, Torino, Italy; 3 Center for Circadian and Sleep Medicine, Department of Neurology, Feinberg School of Medicine, Northwestern University, Chicago, IL USA; 4 Technogel Italia S.r.l., Pozzoleone (VI), Italy; 5 Psychiatric University Clinics, University of Basel, Basel, Switzerland

**Keywords:** Physiology, Translational research

## Abstract

Substantial evidence suggests that the circadian decline of core body temperature (CBT) triggers the initiation of human sleep, with CBT continuing to decrease during sleep. Although the connection between habitual sleep and CBT patterns is established, the impact of external body cooling on sleep remains poorly understood. The main aim of the present study is to show whether a decline in body temperatures during sleep can be related to an increase in slow wave sleep (N3). This three-center study on 72 individuals of varying age, sex, and BMI used an identical type of a high-heat capacity mattress as a reproducible, non-disturbing way of body cooling, accompanied by measurements of CBT and proximal back skin temperatures, heart rate and sleep (polysomnography). The main findings were an increase in nocturnal sleep stage N3 (7.5 ± 21.6 min/7.5 h, mean ± SD; p = 0.0038) and a decrease in heart rate (− 2.36 ± 1.08 bpm, mean ± SD; p < 0.0001); sleep stage REM did not change (p = 0.3564). Subjects with a greater degree of body cooling exhibited a significant increase in nocturnal N3 and a decrease in REM sleep, mainly in the second part of the night. In addition, these subjects showed a phase advance in the NREM-REM sleep cycle distribution of N3 and REM. Both effects were significantly associated with increased conductive inner heat transfer, indicated by an increased CBT- proximal back skin temperature -gradient, rather than with changes in CBT itself. Our findings reveal a previously far disregarded mechanism in sleep research that has potential therapeutic implications: Conductive body cooling during sleep is a reliable method for promoting N3 and reducing heart rate.

## Introduction

Mammals sleep primarily during the trough of their circadian core-body temperature (CBT) rhythm. In humans, the daily time course of CBT is associated with different sleep measures, such as sleep propensity, sleepiness, sleep onset latency or the amount of rapid eye movement sleep (REM)^1–4^. Besides the endogenous regulation of the circadian CBT rhythm, so-called exogenous masking effects also influence CBT. These effects commonly result from daily activities like exercise and rest, nutrition, digestion, or fasting^5^, or even from regular bedtime behaviors like lying down and turning the lights off, and they can also affect sleep^6^.

The circadian down regulation of CBT in the evening is promoted by convective heat transfer from the core to distal skin regions (e.g. hands, feet) resulting from vasodilation and opening of their arteriovenous shunts, while the opposite occurs in the morning CBT increase^7–9^. Not only is the endogenous circadian pattern related to sleepiness and sleep induction, but also skin warming, particularly of the distal regions, leads to a reduction in CBT and a shorter sleep onset latency^10–14^. In contrast to smaller animals, in which CBT decreases during NREM (including slow wave sleep, N3) and increases during REM, humans exhibit no significant alterations of CBT with respect to NREM-REM-sleep cycles^8,15,16^; instead, CBT follows a slow decrease of maximally about 0.3 °C during normal nocturnal sleep^17–19^.

The significance of this CBT decrease for sleep stage changes during nocturnal sleep has not yet been systematically studied. Experimental manipulation of the nocturnal decline in CBT by body cooling during sleep is challenging because any temperature changes beyond the tolerable range can disturb sleep, cold environments are especially disturbing^20^. Successful past examples of thermal intervention have used changes in ambient temperature and humidity, bedding and bedclothes, or mattress type, amongst others^21–25^. In recent years, we have shown that a cooling high heat capacity (HM) mattress can increase conductive body heat loss and N3 without disturbing sleep^26–28^. However, a comprehensive data analysis encompassing all three studies was not conducted, thus lacking the necessary statistical power for a consistent multivariable analysis. Such an analysis would have been essential to effectively and reliably identify the crucial thermophysiological factors responsible for the changes observed in sleep stages. N3 has been regarded as crucial for various processes, such as sleep pressure, endocrine regulation, learning and memory formation, and glymphatic clearance^29^. N3 decreases with age and its absence has been considered a biomarker of cognitive decline^30^. Therefore, potential ways to increase N3 are of high relevance and an area of ongoing research.

The main aim of the present study is to show whether a large decline in CBT during sleep can be related to an increase in slow wave sleep (N3). Therefore, special emphasis was placed on the analysis of the interindividual differences of the mattress induced effects (“heterogeneity of treatment effect”), which can provide insights into the relationships between changes in body temperatures and changes in sleep stages.

## Results

The present data analyses focus on the relationship between body temperature changes and changes in sleep stages occurring during sleep on the cooling mattress. We analyzed data from three study centers using the same experimental protocol. The main aim is to show how mattress induced body cooling during sleep can be related to changes in N3.

### Changes in temperatures and heart rate during sleep initiation

**Figure 1:**
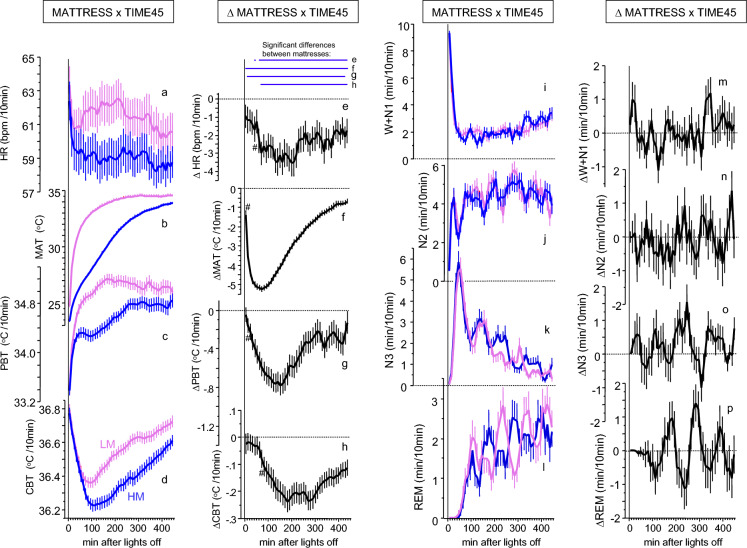
HM induced effects on temperatures, heart rate and sleep stages. Left panels (**a**–**h**): Time courses of temperatures and heart rate (mean ± SEM, 45 × 10min intervals; MATTRESS  ×  TIME45, N = 69–71, Table 1) during 7.5h sleep phase of condition HM (in blue) and LM (in pink) (**a**-**d**) and the differences between the mattresses HM and LM (∆MATTRESS x TIME45, in black) (**e**-**h**). Blue horizontal lines on top of the panel in (**e**-**h**) indicate significant HM-LM differences, referring to the figures below (FDR post hoc’s, p < 0.05). ^#^Indicates the first occurrence of a significant (FDR, p < 0.05) difference between HM and LM after lights off. *HR* heart rate, *MAT* mattress surface temperature, *PBT* proximal back skin temperature, *CBT* core body temperature. Right panels (**i**–**p**): Time courses of sleep stages (mean + and/or–SEM, 45 × 10min intervals, N = 72) for condition HM (in blue) and LM (in pink) ((**i**–**l**), MATTRESS x TIME45) and the differences between HM-LM ((**m**–**p**), ∆MATTRESS x TIME45). W + N1, wake + sleep stage N1; N2, sleep stage N2; N3 slow wave sleep; REM, rapid eye movement sleep. All time courses (**a**–**p**), along with their corresponding standard deviations, are depicted in Suppl. Fig. 3.

Sleep on both mattress types produced the characteristic thermophysiological pattern of a CBT decline that lasts about 1.5 h. Briefly summarized, this initial phase includes redistribution of core blood to the periphery after lying down, switching the lights off and sleep initiation^8^. Proximal back skin temperature (PBT) and mattress temperature (MAT) increased rapidly while heart rate (HR) decreased rapidly, followed by a rather slow decrease in CBT (Fig. 1a–d). The detailed analyses of time courses for each study are presented and discussed in Supplemental Material (Suppl. Fig. 1a-d). For nocturnal mean values see Table 1.

**Table 1:** Mattress effects on nocturnal means, SD, min and max values of the LM, the HM and their differences (HM-LM) (see Figs. 1, 2).

	LM					HM				HM-LM			
	N	Mean	SD	Min	Max	Mean	SD	Min	Max	Mean	SD	Min	Max
HR	69	61.48	8.77	42.99	84.16	59.12	8.49	40.99	78.77	− 2.36	3.62	− 11.96	7.35
MAT	71	33.43	0.85	30.29	35.13	30.63	0.95	28.00	33.24	− 2.80	1.05	− 5.08	− 0.10
PBT	71	34.98	0.54	34.42	36.09	34.54	0.62	32.60	35.51	− 0.44	0.61	− 1.84	0.72
CBT	71	36.56	0.19	36.18	37.20	36.41	0.24	35.94	37.45	− 0.15	0.23	− 0.75	0.64
CBT-PBT	70	1.58	0.52	0.62	2.76	1.87	0.58	0.97	3.64	0.29	0.55	− 0.92	1.58
W + N1	72	2.33	1.26	0.27	5.48	2.36	1.25	0.18	5.17	0.03	1.07	− 2.93	1.76
N2	72	4.30	0.86	2.44	6.32	4.18	0.82	0.93	5.87	-0.21	0.76	− 1.94	1.64
N3	72	1.68	0.77	0.39	4.82	1.85	0.77	0.00	4.63	0.17	0.48	− 1.07	1.49
REM	72	1.62	0.60	0.10	2.97	1.56	0.56	0.30	2.64	- 0.06	0.57	− 1.31	1.22

*HR* (bpm) heart rate, *MAT* (°C) mattress surface temperature, *PBT* (°C) proximal back skin temperature, *CBT* (°C) core body temperature, *CBT*-*PBT* (°C) core body temperature-proximal back skin temperature-gradient, *W* + *N1* (min/10 min) wake + sleep stage N1, N2 (min/10 min), sleep stage N2, N3 (min/10 min), slow wave sleep, REM (min/10 min), rapid eye movement sleep (for statistics including effect sizes see Suppl. Table 1a).

**Figure 2:**
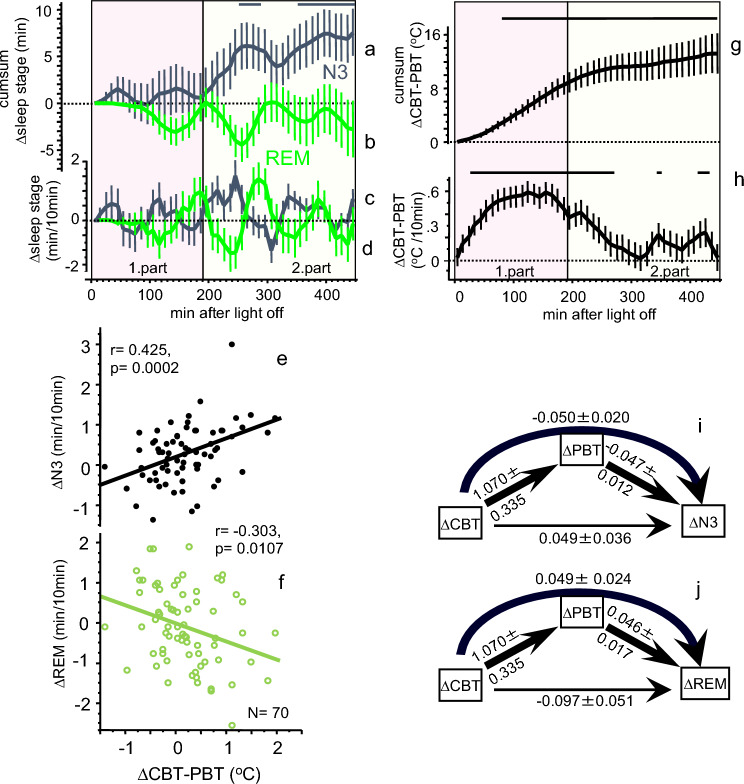
Relationship between changes in temperatures and sleep stages. Upper panel (**a**-**d**, **g** & **h**) From top to bottom, time courses (mean ± SEM; 45 × 10 min intervals, N = 72) of the cumulative sum of ∆N3 (**a**) and the cumulative sum of ∆REM (**b**) are shown, the time courses of ∆N3 (**c**) and ∆REM (**d**) are shown (N3, black curves; REM, green curves). Figure (**g**) and (**h**) shows the corresponding time courses for the CBT-PBT gradient (N = 70; ∆ = HM–LM). The heterogeneity of the treatment effects is depicted in the (**e**,**f**). Significant FDR post-hoc’s per time interval are displayed in horizontal black lines on top of the Figs. 1a,g,h (HM-LM, p < 0.05, ANOVA table see Suppl. Table 1b). The zero (dotted) line represents the LM condition. The pink area indicates the first and the yellow area of the second part of the night. Lower panel (**e**,**f**,**i**,**j**): Significant linear correlation between mattress induced effects in ∆CBT-PBT and ∆N3 ((**e**), r = 0.425, p = 0.0002) and ∆CBT-PBT and ∆REM ((**f**), r = − 0.303, p = 0.0107) for the second part of the sleep phase (190–450 min after lights off). No such correlations were found for the first part of the night (0–190 min after light off, Table 2). Results of the mediation analysis of the mattress induced effects (∆, HM-LM) are shown as path diagrams of the variables ∆CBT, ∆PBT and ∆N3 (**i**), and ∆CBT, ∆PBT and ∆REM (**j**) for the second part of the night (190–450 min). Path coefficients ± SEM are presented beside the path arrows. Significant paths are indicated by thick arrows (p < 0.05), the thin line represents a non-significant path (for statistics see Suppl. Tables 2 & 3). ∆CBT influences ∆N3 and ∆REM mainly via an indirect path through the mediator variable ∆PBT indicated by the curved arrows.

### The cooling mattress reduces nocturnal body temperatures and heart rate

In contrast to the described pattern above during the first 1.5 h after light off, the cooling mattress -induced effects (HM-LM, difference between values measured in sleep on the HM and on the LM) led to a slow reduction of all body temperatures and of HR (Fig. 1e–h; see Suppl. Table 1a). The decline rates of all variables were different, as evidenced by the order of occurrence of significantly declined values (p < 0.05 indicated by # in Fig. 1e–h); and in the occurrence of their minima, which follow the same rank order (Fig. 1e–h). A strong interconnection between changes in CBT and HR was found, as supported by mediation analysis, which showed a significant and consistent relationship between the decrease in CBT and HR, irrespective of the proximal skin temperature (Suppl. Table 4). Our results suggest that the nocturnal reduction in CBT associated with the use of the cooling mattress is attributed to the conductive heat transfer from the core body to the nearby skin on the back and ultimately to the mattress. These effects were observed only during the first part of the night when the most significant heat exchange occurs after lying down and sleep induction (Suppl. Table 4).

### Sleep stage time courses during nocturnal sleep

Sleep stages in both conditions occurred in their characteristic distributions (Fig. 1i–l) with inverse time courses of REM and N3. Compared with the regular mattress (LM), sleep on the cooling mattress (HM) showed an additionally inverse pattern of REM and N3 along the non-REM (NREM) – REM sleep cycle (detailed analyses see below). (see Suppl. Materials for detailed results and discussion). For nocturnal mean values see Table 1.

### The cooling mattress induces a selective increase in nocturnal mean value of N3

Sleep on the HM was associated with a significant increase in the nocturnal mean value of N3 (7.5 ± 21.6 min/7.5 h; p = 0.0038, d = 0.347; Table 1 & Suppl. Table 1a). This increase was also found as % value of total sleep (HM-LM: 1.93 ± 5.02%; F(1, 71) = 10.638, p = 0.0017, d = 0.384).

All other sleep stages did not show any statistically significant differences between the HM and the LM conditions, proving the robust and selective increase in N3 (see Suppl. Table 1a and Suppl. Fig. 2).

### The cooling mattress induces subtle changes in the nocturnal time course of N3 and REM

Sleep stages showed no significant temporal modulation by the cooling intervention over the night. This may indicate that the HM-effects are either homogeneously distributed throughout the night, or that the differences between HM and LM are simply too small to reach statistical significance in a MATTRESS (HM vs. LM) x TIME45 (45 × 10 min intervals) -interaction term (see analyses below). We performed a cumulative sum analysis to statistically record possible small differences in the HM-induced effects on sleep stages over time: Only ∆N3 revealed a significant cumulative sum -time course, with significant increases occurring from 240 min after lights off that lasted, with a short interruption, until the end of the sleep phase (FDR post-hoc’s, MATTRESS x TIME45; see top of Fig. 2a, Suppl. Table 1b).

The systematic increase in the cumulative sum of ∆N3 can only be observed for 190-450 min after lights off, suggesting that a relation between the HM-induced changes in body temperatures and N3 may manifest in the second part of the night. In addition, a superimposed inverse rhythmic pattern can be seen in the time courses of ∆N3 and ∆REM (Fig. 2a–d), which could very likely be related to the NREM-REM sleep cycle. All temperatures decline after lying down and switching the lights off, with CBT reaching the latest minimum at 190 min, which could indicate the end of maximum conductive body heat loss. After 190 min, a slow increase in all temperatures occurs (Fig. 1). For further analyses, we therefore divided the nocturnal time course into a first (0–190 min) and second part (190–450 min after lights off) of the night. Highest significant correlations were found between an increased inner gradient of CBT- proximal back skin temperature (∆CBT-PBT) and increased ∆N3 (Fig. 2e) and decreased ∆REM (Fig. 2f), both selectively in the second part of the night. Similarly, however less pronounced, decreased ∆PBT was correlated with increased ∆N3 and decreased ∆REM (Table 2).

**Table 2:** Correlation matrix ∆body temperatures, ∆heart rate, ∆N3 and ∆REM.

	N	∆N3						∆REM					
		0–190 min			190–450 min			0–190 min			190–450 min		
		r	p-value	r^2^	r	p-value	r^2^	r	p-value	r^2^	r	p-value	r^2^
∆CBT	71	− 0.0146	0.9038	0.000	0.0295	0.8007	0.001	− 0.1852	0.1220	0.034	− 0.1234	0.3054	0.015
∆PBT	71	0.0345	0.7753	0.001	− **0.3951**	**0.0007**	0.156	− 0.2072	0.0830	0.043	** 0.2379**	**0.0455**	0.057
∆CBT-PBT	70	− 0.0265	0.8278	0.001	** 0.4246**	**0.0002**	0.180	0.1398	0.2483	0.020	− **0.3032**	**0.0107**	0.092
∆HR	69	− 0.0224	0.8553	0.001	0.0281	0.8187	0.001	− 0.1685	0.1663	0.028	− 0.2170	0.0733	0.047

Bivariate correlations of the effects of the HM mattress – the LM mattress (HM-LM) of ∆ body temperatures and ∆HR with ∆N3, and ∆REM for the first (0–190 min) and second (190–450 min) part of the night. r, correlation coefficient; r^2^, coefficient of determination = effect size (small effect size, r^2^ = 0.01; medium effect size, r^2^ = 0.09; large effect size, r^2^ = 0.25^31^).

Significant values are in bold.

### Changes in inner heat conduction during sleep are linked to changes in sleep stages

To better understand the causal sequence between the independent (body temperatures) and the dependent variables (sleep stages), we performed mediation analyses. Individual variations of ∆CBT exhibit their influence on both, ∆N3 and ∆REM, mainly by an indirect path via the mediator variable PBT, as indicated by significant indirect paths from ∆CBT → ∆PBT → ∆N3 and ∆CBT → ∆PBT → ∆REM (Suppl. Tables 2, 3). No significant direct paths from ∆CBT → ∆N3 or ∆CBT → ∆REM occurred, neither were any found for the first part of the night (Suppl. Tables 2, 3). The CBT-PBT gradient represents a simple measure of the amount of inner heat flow through the body: The greater the difference between CBT and PBT, the more heat flows from the core to the skin region directly contacting the mattress, e.g. a larger CBT-PBT -gradient leads to larger inner heat flow.

To summarize, compared to sleep on the LM, sleep on the HM was associated with significant reduction in temperatures and HR, as well as with elevated levels of N3. Comparison of the temperature time courses shows that body heat loss during sleep on HM occurs conductively. The maximum of the HM-induced conductive body heat loss is observed at around 190 min which coincides with the beginning of the HM-induced increase of N3.

### Slow nocturnal body cooling is related to sleep stage changes within the sleep cycle

**Figure 3:**
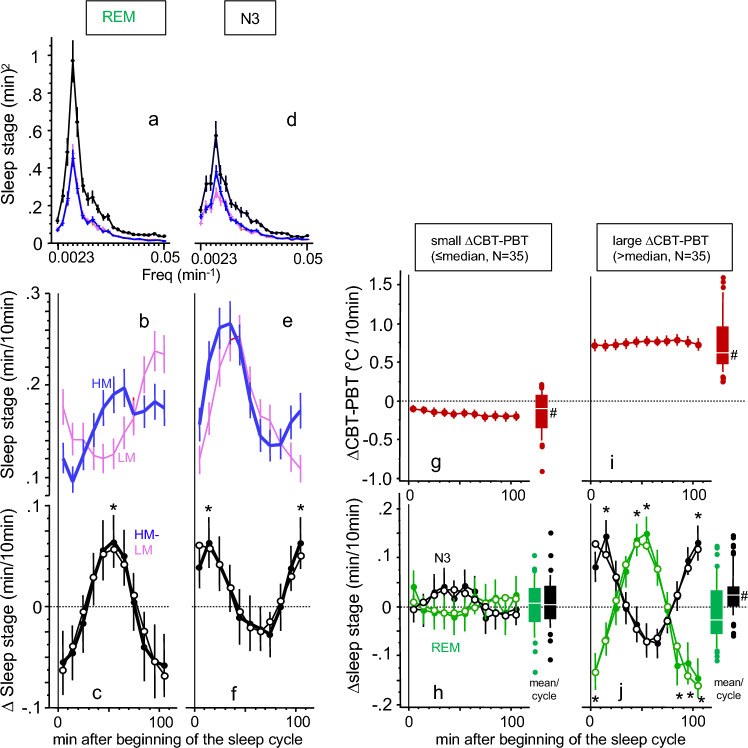
Temperature changes lead to modifications of the sleep cycle. Left panel (**a**,**d**). Power spectra of the Fourier-transformed nocturnal time courses (44 × 10 min intervals, see Fig. 1) of sleep stage REM (**a**) and N3 (**d**) are shown for mattress condition HM (blue curves), LM (pink curves) and HM-LM (black curves). Frequencies are expressed as 1/period (min^-1^, abscissa), spectral power (squared amplitude) is expressed as (min/10 min)^2^ (ordinate) (N = 72, mean ± SEM). Left panel (**b**,**e**). The time courses of REM (**b**) and N3 (**e**) are shown over the averaged 110 min sleep cycle (N = 72, mean ± SEM; 10 min intervals) for HM (blue curves) and LM (pink curves). Significant differences between HM and LM see in Figure (**c**) and (**f**). Left panel (**c**,**f**). HM-LM (∆) values for REM (**c**) and N3 (**f**) is shown over the averaged sleep cycle (N = 72, mean ± SEM; 11 × 10 min intervals, statistics see (**b**) and (**e**)). The zero (dotted) line indicates the LM condition. Significant differences at a given time point between HM and LM are indicated by asterisks (FDR post-hoc’s, p < 0.05). Cosine fit curves for N3 (**c**) and REM (**f**) are superimposed (open dots). Right panel (**g**,**i**). Time courses of ∆CBT-PBT is depicted over the averaged sleep cycle (N = 70, mean ± SEM; 11 × 10 min intervals, ocher colored curves)**.** The sample is median splitted in individuals showing a small ((**g**), left) or large ((**i**), right) HM-LM difference in nocturnal mean values of ∆CBT-PBT. The zero (dotted) line represents the LM condition. The Box and Whiskers plots on the right side of Fig. 3(**g**) and (**i**) show the range of all data points of the mean sleep cycle values (center white line: median; box limits: upper and lower quartiles; whisker limits: 10th and 90th percentile; points: outliers). ^#^Indicates significant differences between HM and LM, p < 0.05, see Suppl. Table 6. Right panel (**h**,**j**). Similar to Figure (**g**) and (**i**) the ∆CBT-PBT median splitted sample is shown for ∆REM (green curves) and ∆N3 (black curves) over the averaged 110 min sleep cycle. Asterisks indicate significant differences between HM and LM at a certain time point ((**j**), FDR post hocs, p < 0.02). The zero (dotted) line represents the LM condition. Cosine fit curves for the median divided subgroups are superimposed (open dots). The Box and Whiskers plots on the right side of Figure (**h**) and (**j**) show the range of all data points of the mean sleep cycle values (center white line: median; box limits: upper and lower quartiles; whisker limits: 10th and 90th percentile; points: outliers). ^#^Indicates significant differences between HM and LM, p < 0.05, see Suppl. Table 6.

In addition to the significant linear association between changes in inner heat conduction (∆CBT-PBT) and changes in ∆N3 and ∆REM (see Fig. 2e,f), a superimposed cyclical antiphase distribution of ∆N3 and ∆REM is apparent, suggesting a potential systematic modulation of the NREM-REM sleep cycle (Fig. 2a–d). To localize potential changes of N3 and REM within a sleep cycle, we performed a sleep cycle analysis in two steps. First, the period of the average NREM-REM sleep cycle was determined using Fourier transformation of the nocturnal time-courses of all sleep stages after omission of the first 10 min after lights off (44 × 10 min-time intervals). We found at a frequency of 0.0091 (min^-1^, equivalent to a period of 110 min) a maximum spectral power for HM and LM in N2 (data not shown), N3 (Fig. 3d), and REM (Fig. 3a), but not in W + N1 (exponential decline, data not shown). Similarly, HM-LM exhibited the same findings (Fig. 3a,d). Based on the findings of the spectral analyses, sleep stage data of the 440 min nocturnal sleep phase was folded (averaged) over four sleep cycles (factor CYC) with a period length of 110 min (factor TIME11). Despite the heterogeneity in the data with respect to sleep cycle distribution and between the three studies, we found strong evidence about where in a sleep cycle the HM-induced effects are located. Figure 3c and f depict the markedly different distribution of REM and N3 over the NREM-REM sleep cycle for both mattress types: Significant differences between HM and LM (indicated by asterisks in Fig. 3c and f) were found for REM at 50–60 min after the beginning of the sleep cycle, and for N3 at the beginning and the end of the sleep cycle (time interval: 10–20 min and 100–110 min). Increases of both, REM (Fig. 3c) and N3 (Fig. 3f) take place in the ascending phase of their sleep cycle rhythm (Fig. 3c and f). To statistically substantiate these findings, we performed cosine fit analyses. The acrophase of ∆REM was found in the middle of the sleep cycle and that of ∆N3 at the beginning of the sleep cycle (for statistics see Suppl. Table 6). The phase difference between ∆N3 and ∆REM was 44.5 ± 17.6 min (p < 0.0001, d = 2.53; Ref.^31^) indicating an almost inverse pattern and confirming the subjective visual impression of the data.

To further substantiate our findings of the effects of increased inner heat flow by sleep on the HM (∆CBT-PBT) on the distribution of ∆N3- and ∆REM during the 110 min—NREM-REM sleep cycle, we performed a median split of the sample by ∆CBT-PBT values. Individuals with small nocturnal increases in the CBT-PBT gradient exhibited no significant changes during the sleep cycles in ∆N3 and ∆REM (Fig. 3g and h), while subjects with large nocturnal increases in ∆CBT-PBT showed large (significant) changes in the sleep stages (Fig. 3i and j) (for statistics see Suppl. Table 6). Moreover, the distribution of individuals undergoing small or large changes in ∆CBT-PBT did not show a significant difference among the three study populations (STUDY vs. MEDIAN, contingency table, [G^2^(df = 2) = 4.341, p = 0.1141]). This suggests a similar distribution of subjects' temperature sensitivity to the cooling mattress HM across studies. Additional cosinor analyses revealed a maximum HM-LM value (acrophase) of ∆N3 at 8.2 ± 20.1 min after beginning of the sleep cycle, and at 54.3 ± 15.8 min (in the middle of the sleep cycle) for ∆REM (cosine fitted curves are superimposed in Fig. 3, for statistics see Suppl. Table 6). The maxima of the HM-induced effects are located clearly before the peak of N3 and REM in condition LM (see Fig. 3b,e), or, in other words, in the rising part of the sleep cycle rhythm. In relation to ∆N3, the sleep cycle of ∆REM is phase delayed by 46.1 ± 3.5 min (p < 0.0001, d = 13.2^31^) (statistics see Suppl. Table 6) (Fig. 3j).

## Discussion

In both mattress conditions MAT and PBT increased immediately after lights off; HR declined fast, while CBT declined slowly (Fig. 1a–d). This pattern, lasting about 1.5 h, is well described and can be explained as a response to lying down, turning the lights off and relaxation during sleep initiation, resulting in reduction in sympathetic tone, melatonin release, and blood redistribution from the core to the shell, especially to distal skin regions^8,26–28^. Vasodilatation in distal skin regions has repeatedly been shown to promote the rapid onset of sleep^10–14,32^, and is therefore an important factor for the onset of the sleep phase.

The initial, fast physiological changes at sleep onset are superimposed by the slow cooling effect of the HM, which reduces all temperature variables and HR below the level of LM, with the strongest and fastest changes occurring in ∆MAT, followed by ∆PBT and ∆CBT (Fig. 1e–h). This rank order reflects the increased heat uptake of the HM mattress, which draws heat from the overlying proximal skin, resulting in a decrease in CBT. Statistical evidence for this interpretation was found through mediation analysis. A significant indirect effect (path) of body heat loss from the core via the back skin to the mattress could be demonstrated for the first part of the night. Additionally, in both parts of the night, a significant direct pathway from the core to the back skin was observed. The gradient between CBT and PBT (CBT-PBT) is therefore a measure of inner heat conduction and reflects the amount of heat transfer from the body core to the shell.

An interesting relationship was found between the HM-induced changes in CBT and HR. Mediation analyses for the first and second parts of the night showed a significant direct path from ∆CBT to ∆HR, regardless of ∆PBT (see Suppl. Table 4). This finding supports the notion that the HM-induced reduction in CBT may be directly responsible for the changes in HR, which is consistent with the results of several previous studies^33^. Active body heat gain (e.g. by exercise) is an energy consuming process, associated with a higher metabolic rate (increased O_2_-consumption) and HR^34–36^. Conversely, intra-individual circadian changes in HR are directly related to changes in O_2_-consumption and (after a delay of about 100 min) to CBT^9^. Furthermore, sinus node activity is directly corelated to temperature changes, and HR changes closely follow changes^34^. Whether only one or several of these mechanisms are responsible for the present findings remains to be clarified. Future studies to investigate whether decreased heart rate is associated with decreased nocturnal blood pressure are needed and could have important therapeutic implications.

Sleep stage distribution followed the characteristic pattern in all three studies (Suppl. Fig. 2). Study 1 had the highest amounts of N3 and REM, while study 2 showed the lowest values, reflecting differences in sleep efficiency at different age. In contrast, all studies consistently and selectively showed a similar increase in sleep stage N3 on the cooling mattress HM^26–28^ (mean increase by HM: + 9.90% of total sleep stage N3), indicating that the increase in ∆N3 is not simply a rebound phenomenon to increased nocturnal waking time, or a consequence of the reduction of another sleep stage. In other words, sleeping on the cooling mattress HM did not disturb sleep (Fig. 1). The detailed temporal evolution of the HM-induced effects on N3 and REM is illustrated in Fig. 2. In the second part of the night, small HM-induced increases accumulate to a significant increase in ∆N3, and to a smaller decrease in ∆REM (n.s.). ∆N3 follows a cyclic pattern, suggesting a modulation by the sleep cycle, whereas the HM-induced changes in body temperatures and HR follow a pattern with minima occurring in the first part of the night (Fig. 1).

Bivariate analysis of all HM-induced changes in body temperatures and HR for the first and second part of the night showed that ∆N3 and ∆REM were selectively correlated to ∆PBT and ∆CBT-PBT in the second part of the night (Table 2). Additional mediation analysis (Fig. 2i and j) revealed that changes in ∆CBT did not have a direct effect on the ∆N3 and ∆REM; instead, an indirect effect via changes in ∆PBT (‘mediator variable’) was found. This indirect path corresponds to the gradient of ∆CBT-PBT, which, according to our findings, indicates the flow of body heat from the core to the proximal back skin.

To better characterize the apparent cyclic changes in REM and N3 during the sleep cycles, we determined the mean NREM-REM sleep cycle length (period) by Fourier transforming the data from time-courses of 45 × 10-min intervals (Fig. 3). This data-derived approach has the advantage of not requiring a definition of the beginning and the end of the sleep cycle, which is per se problematic. A consistent sleep cycle length of 110 min was found for N3 and REM for sleep on both mattress types and for the HM-induced effects, suggesting that the NREM-REM sleep cycle length is not dependent on changes in body temperature to the extent seen in this study. The mean 110 min-sleep cycle length is in line with a recent published wrist activity data-derived analysis^37^, but clearly longer than the 90 min that can often be found in textbooks. Dividing the data into 110-min sleep cycles led to the characteristic pattern with an N3 peak at the beginning and a REM peak at the end of the sleep cycle (Fig. 3). Detailed sleep cycle analysis showed that the increase in N3 and REM occurred selectively in the rising part of the pattern (Fig. 3). In a final analysis using a median split of ∆CBT-PBT, we examined the heterogeneity of treatment effects and found that higher inner body heat flow (i.e., > median of ∆CBT-PBT, indicating cooler PBT relative to CBT) was associated with increased amplitudes of ∆N3 and ∆REM within sleep cycles (see Suppl. Table 6), as well as a significantly higher nocturnal mean value of ∆N3/7.5 h. Conversely, these effects were not observed in subjects with low inner heat flow (see Fig. 3).

Overall, our analyses demonstrate that sleeping on a cooling mattress (HM) enhances the heat flow from the body core to the skin on the back and increases nocturnal N3. Additionally, changes also occurred in the distribution of the sleep stages REM and N3 within the sleep cycle. These changes can be interpreted as a phase advance of the sleep cycle phase for both sleep stages (REM and N3), albeit to varying degrees. It is noteworthy that all the described effects depend on the degree of conductive body heat loss to the back skin.

Investigations using changes in ambient temperature on sleep are difficult to do without disturbing sleep. Still, increased N3 and reduced CBT were found in a study employing a slow declining ambient temperature protocol^25^ and in another study using a rebound mattress -topper^21^, both in line with our findings. Unfortunately, no direct correlations between the temperature changes and sleep stage changes were reported in these studies. However, despite markedly different time courses, a consistent and selective increase in N3 was observed in all studies. The resulting question if and how slow conductive body cooling can also influence the homeostatic regulation of N3, e.g. to improve recovery after sleep deprivation, is subject to future investigation.

**Figure 4:**
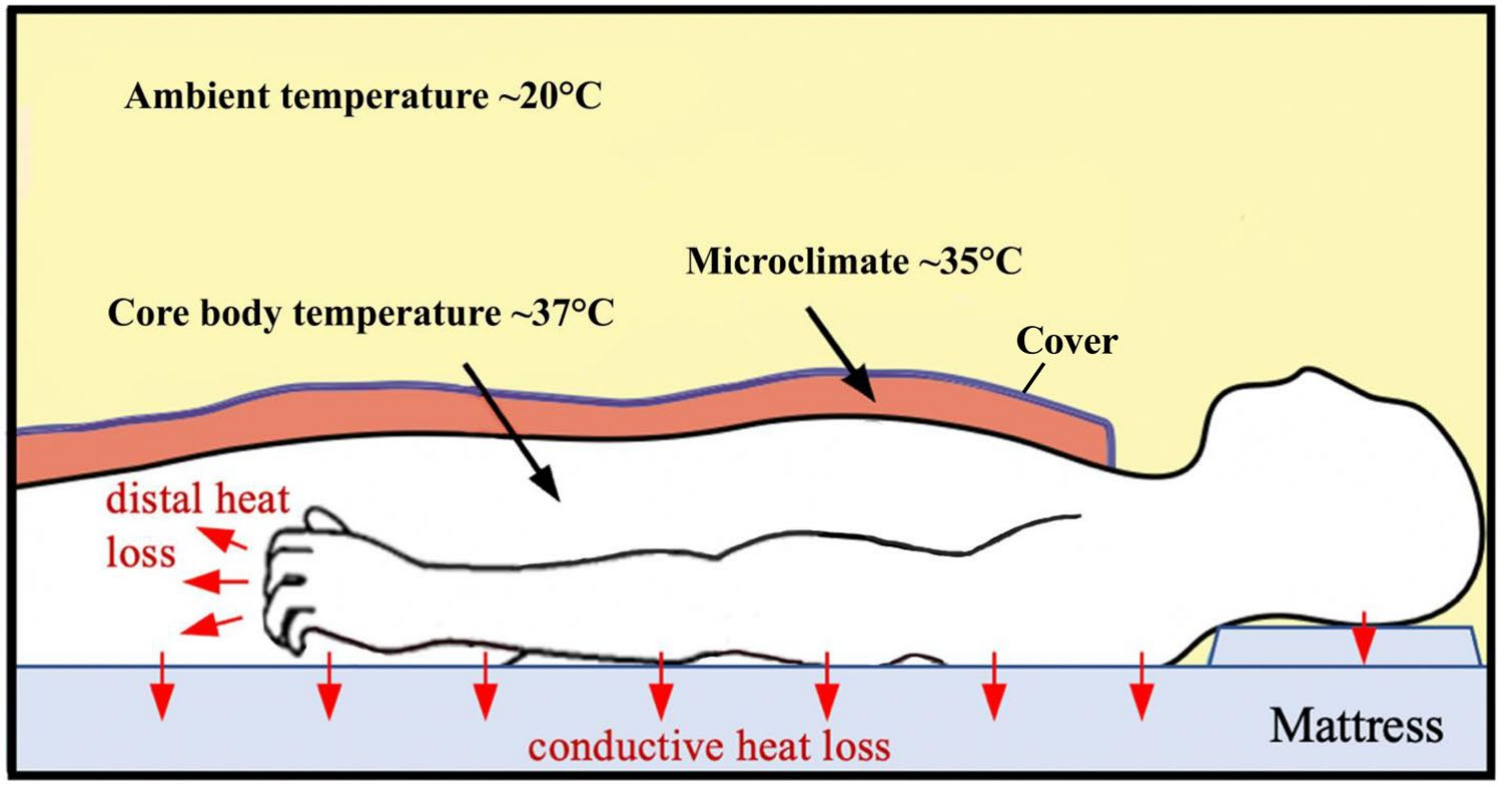
Body temperatures in the human sleep environment. Mammals, including humans, use bedding to create a warm microclimate, which activates hypothalamic mechanisms to initiate convective peripheral heat loss via vasodilation (particularly in distal skin regions, red arrows near the fingers), and sleep. In addition, passive conductive heat loss occurs through heat conduction from the core of the body via the back skin to the mattress (vertical red arrows) leading to increased sleep stage N3 during sleep (extended scheme from Harding 2019 Fig. 2a).

Mammals, including humans, exhibit typical sleep preparation behaviors such as curling up, nest building and use of bedding^38^. These thermoregulatory behaviors generate a microclimate of warmth (31–35 °C) around the skin that enables convective heat loss to vasodilated distal regions, especially in the hands and feet and results in CBT reduction, all of which promotes the rapid onset of sleep^1,12,38^ The warm skin microclimate persists through the sleep phase to maintain a sleep-permissive temperature zone around the body that prevents dangerous cooling. Although only about 20% of the body skin comes into contact with the mattress^39^, the increased heat capacity of the HM led to a reduction in CBT by the order of a circadian amplitude (0.24 °C) without disturbing sleep (Fig. 4).

How can the present results be related to the neurophysiological findings in animals? Sleep is usually divided into two main states, NREM and REM, which are closely coupled to changes in brain temperature (and in CBT) in small laboratory animals^40–47^. In addition to the circadian time course of body temperatures, these animals show a gradual decrease of brain temperature (and CBT) of about 0.2 °C during the transition from wakefulness (and REM) to NREM and vice versa^40,41,43–46^. Humans however exhibit no systematic changes in CBT^2,15^, brain temperature^16^, skin temperatures^8,15^ and heat production^48,49^, with NREM-REM cycles. It therefore seems probable that any temperature changes with NREM-REM cycles are either not present, or much less pronounced in species like humans, with large body size and small specific metabolism. Compared to small laboratory animal, humans exhibit a rather slow reduction of total nocturnal energy expenditure and body temperatures during sleep^8,50^. Nevertheless, the fact that no temperature changes were observed across the human NREM-REM cycle does not rule out the possibility that peripheral temperature changes can induce alterations in sleep stages.

Our novel finding that CBT does not act directly on sleep stages, but that changes in sleep are dependent on PBT is consistent with the notion that the body regulates heat flow including peripheral signals, rather than CBT alone^4,51^. This at least partially supports the idea that thermal afferents from proximal back skin and from the body core are sensed in the preoptic anterior hypothalamus, and act as a determinant of the occurrence of sleep stages^4^. However, our findings reveal in addition a temporal dissociation between the slow HM-induced changes in body cooling and the sleep stage changes that occur in accordance with the sleep cycle. The reason for this temporal discrepancy needs to be further investigated.

As has been observed in laboratory animals, e.g. rats, sleep reduces thermoregulatory activity, whereby the inhibition is less extensive in N3 than in REM^52^. In humans, CBT is usually regulated between thermo-effector thresholds, which are subject to circadian oscillations governed by the SCN. Sleep expands the inter-threshold range, and/or reduces autonomic heat and cold defense mechanisms. The widest inter-threshold range is observed during the circadian trough, which typically coincides with the human sleep phase^53^ when the sympathetic nerve activity is reduced^54^. The results of this study using a thermal intervention in sleep are in line with these previous observations.

This study has several limitations. Firstly, no pre-sleep recordings of HR and temperatures to determine the precise changes occurring after light off were performed. It can be assumed that the values in CBT and HR before lights off would be higher than the values in the first 10 min after that. Secondly, no uniform EEG spectral analyses were performed in Studies 2 and 3, and none in Study 1. Future studies are needed to investigate whether the present findings of increased N3 will be reflected in an increase in delta- power and/or energy. Moreover, sleep scoring was performed by different experts at the respective sleep centers, potentially introducing inter-rater bias. Also, we did not measure sweating rate and brain temperature which could have provided more information about thermophysiological changes during the sleep cycle. Finally, it remains to be conclusively studied how the interaction of age, sex and BMI affects the HM-induced effects on sleep stages, temperatures, and HR, which will require a larger sample.

To summarize and conclude, in this three-center study of thermal intervention in sleep, we show that gently lowering nocturnal body temperatures increases N3 and decreases heart rate. These effects were uniform despite the three heterogenous study samples (age, sex, and BMI). The path and extent of body heat loss could be determined based on nocturnal temperature measurements. The PBT-CBT-gradient provides a reliable measure for inner heat conductance and predicts the increase in N3. In addition, slow nocturnal body cooling also induced a significant phase advance of the N3 and REM patterns within the sleep cycle. Nocturnal conductive body temperature reduction represents a disregarded mechanism in sleep, that holds relevant potential for further basic research as well as for therapeutic applications. Future research is needed to determine if and how much the observed effects can be increased and to understand who could benefit most from a potential therapeutic application.

## Materials and methods

### Study subjects

The underlying studies have been conducted in accordance with the Declaration of Helsinki and each study was approved by the local ethics committees (see Refs.^26–28^). The participants received detailed information on the study purposes and execution and gave written informed consent. In general, all individuals were “normal sleepers”, had no prior medical disorders, and had not recently participated in any other trials. Detailed subject recruitment and inclusion procedures are reported in the original publications. Study 1 consisted of 15 healthy young males with mean age 26.5 ± 1.4 (25–30) years and mean BMI 22.3 ± 1.4 (18.9–24.6) kg/m^2^. Study 2 consisted of 33 healthy male individuals aged 46.2 ± 4.0 (40–55) years with BMI 25.2 ± 1.8 (21.7–29.0) kg/m^2^. Study 3 individuals consisted of 24 healthy post-menopausal women aged 62.5 ± 8.3 (49–75) years with BMI 25.5 ± 3.4 (20.9–33.9) kg/m^2^. Study 1’s mean age and mean BMI are significantly (p < 0.0005) lower than those of the other two studies, while mean age of study 3 is significantly (p < 0.0001) higher than that of the other two studies (ANOVA). Unfortunately, the necessary overlap of the study distributions of age and BMI are too small for reliable analyses of the interaction age x BMI for each variable. The influence of sex cannot be reliably separated from age and BMI either. Total number of subjects (study 1–3, N = 72).

### Study design

This study is based on data from three independent samples which were investigated using the same thermal intervention protocol in Torino (“study 1”; ref. ^27^), Berlin (“study 2”; Ref.^26^), and Chicago (“study 3”; Ref.^28^). All three studies used the same mattress-based temperature intervention for the controlled reduction of sleep temperature on a healthy adult sample. The three studies used a randomized, blinded crossover design. Each subject slept on a (cooling) high-heat capacity mattress (HM) and a (regular) low-heat capacity mattress (LM) on two nights, separated by a one-week interval. The order of the mattresses was counterbalanced in each study. Measurements of CBT, proximal back skin temperature (PBT), mattress temperature (MAT), and heart rate (beats per minute; bpm) were recorded in parallel with full polysomnography (PSG) under controlled sleep laboratory conditions. The recording of all measurements began at lights-off and ceased recording at lights on (total time after lights-off was 450 min for everyone).

### Mattress properties

Temperatures on the HM were compared to the control condition, which used a regular memory-foam mattress (LM). All studies used the same HM, which consisted of a foam core coated in polyurethane material (Technogel, Italia S.R.L., Vicenza, Italy). This coating increased the density and heat capacity of the top 2 cm of the HM (HM: approx. 94.0 kJ/°C; LM: approx. 10.8 kJ/C at 23–35 °C).

### Temperature measurements

The three studies performed comparative temperature (CBT, PBT and MAT) recordings using the same set of temperature sensors and measurement locations. CBT was measured every 15 s using ingestible, telemetric capsule sensors (VitalSense Core Temperature Capsule, Hidalgo Ltd., Cambridge, UK) at 0.01 °C resolution. PBT and MAT were measured once a minute using wireless temperature sensors (“iButton” DS 1922L, Thermochron iButtons; Maxim, Dallas) at 0.0625 °C resolution. Sensors were affixed to the mattress and skin using thin, air-permeable, adhesive surgical tape. Proximal back skin sensors were placed on the back of both shoulders and on the spinal cross between them. MAT was recorded using 5 iButtons placed in a T-shape under the fitted sheet. In all three studies, room temperatures were controlled at 21–23°. For a more detailed description of the temperature measurements and protocols used, see the original publications^26–28^.

### Polysomnography recording and scoring

In each condition (HM and LM), full video polysomnography (PSG) recordings were performed in sleep laboratories using a standard montage. Procedures followed the American Academy of Sleep Medicine (AASM) protocol and are described in the original publications. PSG data were blind scored by a sleep specialist according to AASM criteria. Of note, the three studies used certified PSG equipment from different manufacturers (study 1: Grass Telefactor, Astro-Med Inc., West Warwick, RI 02893 USA; study 2: Embla Inc., Broomfield, CO, USA; study 3: Neurofax EEG-1100, Nihon Kohden, Japan).

### Heart rate analysis

Heart rate (HR) was derived from the PSG data based on the ECG inter-beat interval. HR was identified automatically, and artifacts were eliminated with visual inspection. Study 1 and 2 applied RemLogic-software (Ver. 3.4, Embla Inc., Broomfield, CO, USA) and study 3 used PRANA -software (PhiTools, Strasbourg, France).

### Data analysis

For statistical time-course analyses, temperatures and sleep stages were aggregated into 10 min. time segments. Differences in sleep stages and temperatures between the HM and LM were examined temporally across the night in 10 min intervals. ANOVA mixed effects models with repeated factors TIME45 (45 × 10 min), factor CYCLE (4 sleep cycles), TIME11 (11 × 10 min) and MATTRESS (HM vs. LM) and one grouping factor, STUDY (study 1, 2 and 3), and subjetcs (as random factor), were performed with mixed effects models using the package “nlme” in R^55^. Post-hoc comparisons were calculated using the false discovery rate (FDR, Benjamini–Hochberg procedure) adjusting p-values for multiple comparisons^56^. Group data are presented as mean ± SD. The effect sizes of ANOVA -derived effects are expressed as eta squares (η^2^) calculated using the R package “rstatix” (small η^2^ = 0.01; medium η^2^ = 0.06; large η^2^ = 0.14; Ref.^31^). R-squares (r^2^) were used as the effect size measure for linear regression analyses (small effect size, r^2^ = 0.01; medium effect size, r^2^ = 0.09; large effect size, r^2^ = 0.25; Ref.^31^), while Cohen’s d was employed for assessing the effect size of group mean differences (small effect size, d = 0.2; medium effect size, d = 0.5; large effect size, d = 0.8; Ref.^31^).

For statistical analysis of the data in relation to the NREM-REM cyclicity, their period length is required. To estimate the period length of the NREM-REM sleep cycles, the time series (45 × 10 min intervals) of the sleep stages N3 and REM were analyzed by spectral analyses using the function ‘spectrum ( )’ of the statistics program R^57^. This function calculates the individual power density spectrum after linear detrending. Two separate cosine models were used for the subsequent sleep cycle time courses. First, a cosinor model for the population mean (R package ‘cosinor2’) was used to determine the mean coefficients of ∆REM and ∆N3, and to detect the overall significance of the cosinor model (“rhythmicity”). Second, we fitted a cosinor mixed effects model using R package “cosinoRmixedeffects”, which includes random effects for mesor, amplitude and acrophase. This model was applied to calculate the phase differences in sleep cycle time courses between sleep stage ∆N3 and ∆REM.

The chosen approach does not require an individual a priori classification of sleep data into NREM-REM cycles, which is often very difficult to perform.

Mediation analyses were performed using R package “mediation”. The confidence intervals were estimated based on the nonparametric bootstrap method with 1000 simulations.

### Supplementary Information

Supplementary Information.

## Data Availability

All data used in this study are available from the corresponding author K.K. upon request.

## Abbreviations

BMI: Body mass index
CBT: Core body temperature
HM: (Cooling) high heat capacity mattress
HR: Heart rate
LM: (Regular) low heat capacity mattress
MAT: Mattress
N3: Slow wave sleep
NREM: Non-REM sleep
PBT: Proximal back skin temperature
REM: Rapid eye movement sleep
