# Supplementary material for: Enhanced conductive body heat loss during sleep increases slow-wave sleep and calms the heart
Source: Sci Rep. 2024 Feb 26;14:4669. doi: 10.1038/s41598-024-53839-x (PMC10897321; doi:10.1038/s41598-024-53839-x)
Supplement: Supplementary file 1 — Supplementary Information. [file 41598_2024_53839_MOESM1_ESM.docx]

***Supporting information***

**Abbreviations**

BMI, *body mass index;* CBT, *core body temperature;* HM, *high heat capacity mattress;* HR, *heart rate;* LM, *low heat capacity mattress;* MAT, *mattress temperature;* N3, *slow wave sleep*; NREM, *non-REM sleep;* PBT, *proximal back skin temperature;* REM, *rapid eye movement sleep*

1. **Study sample differences in nocturnal time courses of temperatures and HR related to mattress induced effects.**

# In general, time courses of temperatures and HR during sleep revealed the well-known patterns as described in many publications (e.g. ^1^). The initial phase lasted about 1.5 hours and showed the fastest changes in all studies; PBT and MAT increased, while CBT and HR decreased. However, there are pronounced differences between studies, as shown in Suppl. Fig. 1, left panel (STUDY x TIME45, all p<0.0001). In general, study 1 presented significant temperature and HR differences in comparison to studies 2 and 3, while studies 2 and 3 were in many cases similar. Interestingly, mattress-induced effects showed hardly any differences in time courses between the studies, which could be due to insufficient statistical power to detect small differences. Only MAT revealed a significant MATTRESS x TIME45 x STUDY interaction (see below). Detailed description of the results for each variable of the interaction term STUDY x TIME45 is presented below (post-hoc comparisons are based on FDR and given in the Figure legend 1).

**Suppl. Figure 1**

#

**Legend to Suppl. Figure 1. a-d, left panel. Time courses of temperatures and heart rate split by studies (HM and LM pooled)**.

From bottom to top, nocturnal time courses (mean ± SEM, 10 min-intervals) are shown for core body temperature (CBT), proximal back skin temperature (PBT), mattress surface temperature (MAT) and heart rate (HR, beats-per-minute, bpm).

---------------------

All variables exhibited significant STUDY x TIME45 interactions (p<0.0001).

FDR adjusted post-hoc's (p<0.05) tested for all 45x10min time -intervals:

CBT: study1 > study2,3, time interval 10-110min; study1 < study2,3, time interval 190-450min; and study3 > study 2, between time interval 430-450min. 7.5h nocturnal means (±sem) for each study, are displayed in a bar chart next to each time course (main effect: STUDY).

PBT: study1 > study2, time interval 10-20min; study1 > study3, time interval 10-50min; study1 < study2, time interval 140-450min; study1 < study3, time interval 180-450min.

MAT: study1 > study2,3, time interval 30-60min; study1 < study2,3, time interval 260-450min.

HR: study1 < study2,3, time interval 130-450min.

CBT, PBT, MAT and HR showed significant lower values in study1 compared to study2 and 3 (*p<0.05). Time courses of study1 are significantly different from study2 and study3 for all variables.

**Legend to Suppl. Figure 1. e-h, right panel. Time courses of HM -induced effects (HM-LM) in temperatures and heart rate split by studies**.

From bottom to top, nocturnal time courses (mean ± SEM; 10 min-interval, n=70) of CBT, PBT, MAT and HR are shown as differences between the HM and LM condition (the zero line indicates the LM condition).

---------------------

All three-way interactions MATTRESS x TIME45 x STUDY [all F(88,6052)<1.05, p>0.1754]. FDR post-hoc's didn't reveal any significant differences between the studies tested for all 45x10min -intervals (only statistical trend, p<0.1, for MAT: study 1< study 3, time interval 40-80min). The overall nocturnal mean difference between HM-LM of the studies (STUDY x MATTRESS) for each variable is displayed in a bar chart next to each time course. *p <0.05 study 1 < study 3. Despite the large differences in nocturnal time courses between studies, the HM-induced effects are similar for all variables.

CBT (Suppl. Fig 1d).

Compared to studies 2 and 3 (middle aged and elderly individuals), study 1 (young male adults) showed a significantly (p < 0.002) higher CBT at the beginning of the sleep phase (first 10-minute interval; 36.98±0.07°C [study 1] vs. 36.70±0.07°C [study 2] and 36.77±0.05°C [study 3]), and a more pronounced and longer lasting decline in CBT throughout the night. Study 1 CBT was at its minimum 280min after lights-off (36.15±0.04°C), while study 2 and study 3 CBT reached a minimum strikingly earlier, 100min and 90min after lights-off (36.27±0.03°C, 36.25±0.05°C). As a result, CBT in studies 2 and 3 started to rise earlier than study 1. Due to the differences in time courses, CBT in studies 2 and 3 was lower the first 2 hours, but higher the last 4.5h of the night in comparison to CBT in study 1. The overall night analysis (main effect STUDY) revealed a significantly lower CBT in study 1 compared to studies 2 and 3 (∆-0.18°C/7.5h, p<0.0003; Suppl. Fig.1d).

PBT (Suppl. Fig 1c). Compared to studies 2 and 3, study 1 presented significantly higher PBT at the beginning of the night (34.10±0.11°C vs. 33.31±0.10°C [study 2]; and 33.10±0.12°C [study 3]). The elevation lasted 20min compared to study 2 and 50min compared to study 3. Conversely, study 1 exhibited lower PBT values than studies 2 and 3, 2.3h after lights-off. Similar to CBT, the overall night analysis (main effect STUDY) demonstrated a significant lower PBT in study 1 compared to studies 2 and 3 (∆-0.48°C/7.5h, p<0.0007; Suppl. Fig 1c).

MAT (Suppl. Fig 1b). MAT in study 1 presented a similar pattern, increasing faster at the beginning of the night than MAT in studies 2 and 3 (significantly different 20-60min after lights-off). Conversely, MAT in study 1 remained significantly lower during the last 260 minutes of the night. Similar to CBT and PBT, the overall night analysis (main effect STUDY) demonstrated a significant lower MAT in study 1 compared to studies 2 and 3 (∆-0.46°C/7.5h, p<0.05; Suppl. Fig 1b). However, the time course of MAT is significantly modulated by the factor MATTRESS (MATTRESS x TIME45 x STUDY, F(88,5984)=1.920, p<0.0001). Compared with LM, HM reduced MAT stronger in study 1 than in studies 2 and 3 and only around its minimum 30-80min after lights off (Suppl. Fig 1f).

HR (Suppl. Fig 1a).

In comparison to studies 2 and 3, study 1 showed sustained significantly reduced HR values 2 hours after lights-off. Study 1 HR continued to decline to a flat minimum in the second half of the night, while studies 2 and 3 HR showed already a minimum 30-40min after lights-off. Similar to the body temperatures, overall night mean value of HR was significantly lower in study 1 than in studies 2 and 3 (∆-2.4bpm/7.5h, p<0.02; main effect STUDY; Fig.1a).

To sum up, in comparison to studies 2 and 3, study 1, although warmer in the first 100 minutes of the night, presented significantly reduced CBT, PBT, MAT and HR -values in both mattress -types throughout the nocturnal 7.5h -sleep phase.

Study sample differences in nocturnal time courses of temperature and HR in relation to age, sex and BMI.

The previously described heterogeneous study samples differed significantly in the time courses of the measured variables (CBT, PBT, MAT and HR) within both conditions HM and LM (Suppl. Fig1). Unfortunately, due to the study distribution of age, BMI and sex their influences on the data cannot be completely separated (see Methods: study subjects). Nevertheless, it can be deduced with high probability that ‘age’ is decisive for the study differences in the time courses (Suppl. Fig1), despite moderate differences in BMI. Based on the study findings, factor ‘sex’ can be excluded as a main confounding factor. In both conditions HM and LM, study 1 including the youngest individuals (age ≤ 30 years) presented significant differences of temperatures and HR in comparison to studies 2 and 3 (age ≥ 40 years), which were very similar to each other (Suppl. Fig 1a-d). The large differences between study1 and studies 2 and 3 in the apparent phase differences in CBT -nadir of around 200 min cannot be explained by differences in endogenous circadian timing, but rather by different masking effects induced with sleep induction ^2^. The sustained reduction occurred in nocturnal heart rate in younger individuals (Suppl. Fig. 1a) may be due to a greater sleep-induced reduction in metabolic rate (O_2_-consumption) resulting in a sustained reduction in CBT, PBT and MAT (Suppl. Fig. 1b-d). No studies exist to the authors’ knowledge that systematically investigated interactions of the factors age, BMI and sex on nocturnal time courses of CBT, HR and skin and mattress temperatures, and none considered these variables with respect to nocturnal body heat transfer. At least, several previous studies have described differences of nocturnal CBT time-courses in populations of different ages and found an earlier nadir in older individuals, similar to our findings ^3-7^. Within this context it is interesting to mention that older individuals showed a lower CBT at sleep onset (Suppl. Fig 1d), which can be interpreted as a consequence of their lower daytime activity level (sedentary lifestyle and hence lower metabolic rate) ^2,8,9^.

In sum, our data suggest that younger age is probably associated with a stronger reduced nocturnal metabolism during sleep, as reflected in both lower HR and lower body temperatures ^10^.

1. **Study sample differences in nocturnal time courses of sleep stages related to mattress induced effects.**

Time course analysis of sleep stages within both conditions (HM and LM) revealed the characteristic patterns for each study (Suppl. Fig. 2a-d), as described for the combined group (Fig. 1a-d). Inverse time courses of REM and N3 with minima and maxima at the beginning and end of the sleep phase were found. In all three studies the superimposed NREM – REM sleep cycles showed the inverse pattern for REM and N3 within HM and LM. All time courses (except for REM) were modulated by factor STUDY (TIME45 x STUDY; all p<0.0002) (Suppl. Fig. 2a-d).

**Suppl. Figure 2**

**Legend to Suppl. Figure 2. a-d, left panel. Time courses of sleep stages split by studies (HM and LM pooled)**.

From bottom-to-top, time courses (mean ± SEM; 10 min-intervals, N=72) of REM, N3, N2, and W+N1 over the 7.5h sleep phase are shown.

----------------------

All variables exhibited no significant MATTRESS x TIME45 x STUDY -interactions (all n.s.), however, significant STUDY x TIME45 interactions (p<0.0002, except REM n.s.). FDR post-hoc's (p at least <0.05) tested for all 45x10min time intervals: N3: study1 > study2, time interval 40,160,220, 230, 250min; study2 < study3, time interval 40, 110, 160, 170, 200min; N2: study1 < study2; W+N1: study1 < study3, time interval 360, 430-450min; study1 < study2, time interval 100, 160, 190-350, 380-450min; study2 > study3, time interval 20, 30, 100, 130, 160, 190, 200, 230-250, 290-310, 390-410min; Overall means±sem for the entire 7.5h sleep phase of each study are displayed in a bar chart next to each time course (main effect: STUDY): N3 and REM showed significant lower values in study2 compared to study1 and study3 (*p at least <0.05). W+N1 showed significant differences between all studies (*p<0.05) with highest values in study2. N2 revealed no significant differences, n.s. Over the entire sleep phase, more N3 and REM and less W+N1 were found in study2 compared to study1 and 3, indicating a reduced sleep efficiency in study2.

**Legend to Suppl. Fig. 2. e-h, right panel. Time courses of mattress-induced effects in sleep stages split by studies**.

From bottom to top, time courses (mean ± SEM; 10 min-intervals, N=72) of REM, N3, N2, and W+N1 sleep over the 7.5h sleep phase are shown as differences between HM and LM (the zero line indicates the LM condition).

----------------------

No significant interactions were found in any variable with respect to MATTRESS x TIME45 x STUDY, MATTRESS x STUDY, MATTRESS x TIME45 and MATTRESS (all n.s.). No differences were found between HM and LM, even after considering the factors STUDY and TIME45. The bar charts in Suppl. Fig. 2c demonstrate significant reductions in both N3 and REM, and increased W+N1 in study 2 compared to studies 1 and 3 (significant main effect STUDY, p<0.05), leading to a reduced sleep efficiency in study 2 (study 2, N=33: 81.7±1.4% vs. study 1&3, N=39: 87.1±1.0%, p=0.0020). Additionally, the increase of SOL in study 2 (study 2, N=33: 15.6±1.5 vs. study 1&3, N=39: 11.4±0.9, p=0.0171) indicates generally worse sleep of the individuals. For all sleep stages the factor MATTRESS did not reveal any significant influence on the nocturnal time courses of the studies (MATTRESS x TIME45 x STUDY, all F(88,6072)<1.0779, all p>0.2915).

# Detailed description of the results for each variable of the interaction term STUDY x TIME45 is presented below.

REM (Suppl. Fig. 2d). REM time course did not demonstrate a significant TIME45 x STUDY interaction, but showed a significant main effect STUDY with lower mean values in study 2 compared to studies 1 and 3 (∆-0.37min/10min, p<0.002; Suppl. Fig. 2d).

N3 (Suppl. Fig. 2c). N3 revealed a significant TIME45 x STUDY interaction (p<0.0002). The post-hoc analyses showed that mainly study 2 differed from the two others. The reduction occurred predominantly in the second and third non-REM sleep cycle. Study 2 showed a significantly lower overall night mean N3 -value in comparison to studies 1 and 3 (∆-0.60min/10min, p<0.0004; Suppl. Fig. 2c).

N2 (Suppl. Fig. 2b). Despite the significant interaction (TIME45 x STUDY, p<0.0002), N2 post-hoc analyses revealed only one significant difference at time interval 4, with lower values in study 1 than 2. However, N2 did not show any significant differences in overall night means between the studies (Suppl. Fig. 2b).

W+N1 (Suppl. Fig. 2a). In addition to N3, W+N1 is another sleep variable showing significantly different time courses in the three studies (TIME45 x STUDY, p<0.0001). In general, study 1 exhibited the lowest values, followed by study 3 and study 2 the highest, however, the differences between the studies varied over time. In the first about 3 hours, W+N1 was significantly higher in study 2 compared to studies 1 and 3. Subsequently, the W+N1 values in study 3 increased successively, whereby the values in study 1 remaining constantly low and those in study 2 constantly high. Analysis of overall night mean values revealed highest W+N1 values in study 2 and lowest in study 1 (∆0.135min/10min, p<0.0001; Suppl. Fig. 2a). Sleep efficiency [(REM+N1-3)*100/total time lights off] is significantly higher in study 1: 90.1±1.4% than study 2: 82.5±1.3% and study 3: 85.0±1.6% (study 1 > studies 2 and 3, p<0.05; study 2 vs. 3, n.s.).

To sum, our data suggest that middle-aged men generally slept the worst compared to young men and post-menopausal women. However, this finding is not simply reflected in parallel thermophysiological changes. The thermophysiological patterns are very similar in studies 2 and 3 and significantly different from study 1, indicating a certain independency of the thermophysiological and sleep systems.

----------------------------------------------------------------------------------------------------------------

**Suppl. Figure 3**

**Legend to Suppl. Figure 3**

**HM induced effects on temperatures, heart rate and sleep stages**

In order to show the heterogeneity of inter-individual differences in the time courses, standard deviations of the mean values are added (± 1 SD, which includes approximately 68% of all subjects, see also Table 1 and Fig.1).

**Left panels:** Time courses of temperatures and heart rate (mean ± SD, 45 x 10min intervals; MATTRESS x TIME45; N=69-71, Table 1) during 7.5h sleep phase of condition HM (in blue) and LM (in pink) (1a-d,) and the differences between the mattresses HM and LM (∆MATTRESS x TIME45, in black, 1e-h).

**Right panels:** Time courses of sleep stages (mean ± SD, 45 x 10min interval; MATTRESS x TIME45; N=72) for condition HM (in blue) and LM (in pink) (1i-l, MATTRESS x TIME45) and the differences between HM and LM (∆MATTRESS x TIME45, in black, 1m-p).

----------------------------------------------------------------------------------------------------------------

**Suppl. Table 1a.**

Variable MATTRESS TIME45 MATTRESS x TIME45

N DF F p η^2^ DF F p η^2^ DF F p η^2^

HR 69 1,6052 588.584 **<0.0001** 0.016 44,6052 3.1688 **<0.0001** 0.004 44,6052 1.11348 0.25049 0.001

MAT 71 1,6230 19485.0 **<0.0001** 0.629 44,6230 1240.6 **<0.0001** 0.826 44,6230 142.080 **<0.0001** 0.352

PBT 71 1,6230 754.008 **<0.0001** 0.070 44,6320 30.361 **<0.0001** 0.118 44,6230 3.55529 **<0.0001** 0.015

CBT 71 1,6230 716.989 **<0.0001** 0.066 44,6320 40.346 **<0.0001** 0.150 44,6230 3.05505 **<0.0001** 0.013

CBT-PBT 70 1,6141 380.916 **<0.0001** 0.036 44,6141 44.530 **<0.0001** 0.162 44,6141 3.59068 **<0.0001** 0.015

W+N1 72 1,6319 0.15800 0.69102 0.000 44,6319 28.232 **<0.0001** 0.145 44,6319 1.01989 0.43539 0.006

N2 72 1,6319 1.83401 0.17556 0.000 44,6319 7.6620 **<0.0001** 0.049 44,6319 0.70035 0.93323 0.005

N3 72 1,6319 5.24193 **0.02208** 0.001 44,6319 27.485 **<0.0001** 0.153 44,6319 0.94285 0.57991 0.006

REM 72 1,6319 0.00001 0.99999 0.000 44,6319 5.557 **<0.0001** 0.057 44,6319 1.43841 0.03039* 0.010

**Legend to Suppl. Table 1a.**

ANOVA table (mixed effects models) see Figure 1 & Table 1. Significant p-values are bolded.

Abbreviations: HR, heart rate; MAT, mattress surface temperature; PBT, proximal back skin temperature; CBT, core body temperature; W+N1, wake + sleep stage N1; N2, sleep stage N2; N3, slow wave sleep; REM, rapid eye movement sleep; N, number of subjects; DF, numerator and denominator of the degrees of freedom; η^2^ = generalized eta-squared measure of effect size (small η^2^ =0.01; medium η^2^ =0.06; large η^2^ =0.14; ^11^). * We ignored the small changes of MATTRESS x TIME45 in REM since no significant HM-LM values were found (all FDR adjusted post-hoc's, p>0.22665).

**Suppl. Table 1b.**

∆MATTRESS x TIME45

variable DF F p η^2^

cumulative sum ∆N3 (Fig.2a) 44,3124 4.0194 **<0.0001** 0.019

cumulative sum ∆REM (Fig.2b) 44,3124 0.8018 0.8218 0.006

cumulative sum ∆CBT-PBT (Fig.2g) 44,3036 18.722 **<0.0001** 0.073

∆N3 (Fig.2c) 44,3124 1.0656 0.3563 0.014

∆REM (Fig.2d) 44,3124 1.4089 0.0394* 0.019

∆CBT-PBT (Fig.2h) 44,3036 5.5907 **<0.0001** 0.045

**Legend to Suppl. Table 1b.**

ANOVA table (mixed effects models) for HM-LM values (∆), see Legend to Suppl. Table 1a.

*see comment Legend to Suppl. Table 1a. Small effect size (η^2^) =0.01; medium η^2^ =0.06; large η^2^ =0.14; ^11^.

**Suppl. Table 2. Mediation analysis between ∆CBT, ∆PBT and ∆N3 (N=70).**

0-190min 190-450min

indirect effects: mean 95%CI(lower,upper) p-value mean 95%CI(lower,upper) p-value

∆CBT → ∆PBT → ∆N3 4.64e-3(-3.89e-2,5.0e-2) 0.80 **-4.97e-2(-8.99e-2,-2.0e-2) 0.002**

direct effects:

∆CBT → ∆N3 8.47 (-1.15e-1, 5.0e-1) 0.85 4.89e-2(-8.87e-2, 1.2e-1) 0.106

∆PBT → ∆N3 4.91e-3±2.20e-2 (mean±sem) 0.824 **-4.65e-2±1.21e-2**(mean±sem) **0.0003**

∆CBT → ∆PBT **9.461e-1±2.86e-1 0.0015 1.0703±3.349e-1 0.0021**

total effects mean 95%CI(lower,upper) mean 95%CI(lower,upper)

∆CBT →∆N3 1.31e-1 (-9.16, 1.30e-1) 0.75 -8.11e-4 (-5.54e-2, 7.0-2) 0.966

**Suppl. Table 3. Mediation analysis between ∆CBT, ∆PBT and ∆REM (N=70).**

0-190min 190-450min

indirect effects: mean 95%CI(lower,upper) p-value mean 95%CI(lower,upper) p-value

∆CBT → ∆PBT → ∆REM -1.38e-2 (-4.79e-2,1.0e-2) 0.29 **4.91e-2 (8.82e-3,1.10e-1) 0.008**

direct effects:

∆CBT → ∆REM -3.35e-2 (-1.04e-1, 5.0e-2) 0.45 -9.65e-2(-2.17e-1, 0.0) 0.064

∆PBT → ∆REM -1.46e-2±1.20e-2 (mean±sem) 0.226 **4.59e-2±1.73e-2** (mean±sem) **0.0099**

∆CBT → ∆PBT **9.46e-1±2.86e-1 0.0015 1.0703±3.349e-1 0.0021**

total effects mean 95%CI(lower,upper) mean 95%CI(lower,upper)

∆CBT →∆REM -4.73e-2 (-1.07e-1, 2.0e-2) 0.21 -4.74e-2 (-1.50e-1, 4.0e-2) 0.274

**Suppl. Table 4. Mediation analysis between ∆CBT, ∆PBT and ∆HR (N=67).**

0-190min 190-450min

indirect effects: mean 95%CI(lower,upper) p-value mean 95%CI(lower,upper) p-value

∆CBT → ∆PBT → ∆HR 5.372e-2 (-9.00e-1,2.11) 0.48 -4.189e-2 (-1.375,1.45) 0.93

direct effects:

∆CBT → ∆HR **6.12 (2.63,9.62) <0.0001** **7.62(2.71,1.32e1) <0.0001**

∆PBT → ∆HR -1.46e-2±1.18e-2 (mean±sem) 0.2154 -3.95e-2±6.44e-1 (mean±sem) 0.9512

∆CBT → ∆PBT **9.622e-1±2.943e-1 0.0017 1.0616±3.45e-1 0.0031**

total effects mean 95%CI(lower,upper) mean 95%CI(lower,upper)

∆CBT →∆HR **6.65 (3.52,1.00e1) <0.0001** **7.57(2.78,1.23e1) <0.0001**

**Suppl. Table 5. Mediation analysis between ∆CBT, ∆PBT and ∆MAT (N=69).**

0-190min 190-450min

indirect effects: mean 95%CI(lower,upper) p-value mean 95%CI(lower,upper) p-value

∆CBT → ∆PBT → ∆MAT **4.97e-1 (1.01e-1,1.05) 0.006** 1.30e-1 (-3.39e-1,6.80e-1) 0.53

direct effects:

∆CBT → ∆MAT -1.29e-1 (-9.54e-1,6.10e-1) 0.76 5.37e-1(5.63e-1,1.54) 0.31

∆PBT → ∆MAT **5.089e-1±2.262e-1** (mean±sem) **0.0278** 1.21e-1±2.06e-1 (mean±sem) 0.558

∆CBT → ∆PBT **9.758e-1±2.900e-1 0.0013** **1.071±0.339e-1 0.00234**

total effects mean 95%CI(lower,upper) mean 95%CI(lower,upper)

∆CBT →∆MAT 1.35 (-1.35e1,1.10e1) 0.352 6.67e-1(-2.85e-1,1.61) 0.15

**Legend to Suppl. Table 2-5.**

Mediation analyses were calculated separately for the first (0-190min) and second (190-490min) part of the sleep phase [estimated path coefficient with 95% Confidence Interval (95%CI), lower and upper limit, or in Mean±SEM].

**Suppl. Table 2.&3.** Mediation analyses between ∆CBT, ∆PBT and ∆N3. Only for the second part of the night a significant indirect path from ∆CBT via ∆PBT to ∆N3 (Suppl. Table 1) and ∆REM (Suppl. Table 2) was found. No significant total effects and no significant direct path from ∆CBT to ∆N3 and from ∆CBT to ∆REM were found. (see Fig.2i & 2j)**.** For both N3 and REM a significant indirect path from ∆CBT → ∆PBT → ∆N3 or ∆REM was found. ∆PBT is identified as the crucial mediator variable for changes in these sleep stages.

**Suppl. Table 4.** Mediation analyses between ∆CBT, ∆PBT and ∆HR. For both parts of the night a direct path from ∆CBT to ∆HR was found without any significant effects of ∆BPT on ∆HR. ∆CBT affects ∆HR directly without indirect path via ∆PBT.

**Suppl. Table 5.** Mediation analyses between ∆CBT, ∆PBT and ∆MAT. Only in the first part of the night a significant indirect path from ∆CBT → ∆PBT → ∆MAT was found. The significant indirect path provides evidence for the role of conductive body heat loss to the mattress during the first part of the night.

- - - - - - - -

**Suppl. Table 6.**

**Cosine fit analysis of the sleep-cycle patterns of HM induced effects (HM-LM) (mean±SD)**

**(see Figures 3c,f,h&j):**

Mesor: p: d: Amplitude: p: d: Acro.(rad): Acro.(min): p: d: rhythm. p:

**All subjects (N=72, Fig. 3 c&f):**

∆REM -0.0063±0.0649 0.3576 0.097 0.0641±0.0918 <**0.0001** 0.698 -3.2732±1.4373 -57.3±27.0 **<0.0001** 2.125 **<0.0001**

∆N3 0.0171±0.0590 **0.0171** 0.290 0.0436±0.0834 <**0.0001** 0.523 -0.7272±1.9153 -12.7±34.5 **0.0021** 0.368 **<0.0001**

∆N3-REM* 0.0234±0.0556 **0.0009** 0.421 -0.0311±0.1677 0.1233 0.185 2.5456±0.9909 +44.5±17.7 **<0.0001** 2.515 **<0.0001**

**‘≤median’ CBT-PBT (N=35, Fig. 3h):**

∆REM 0.0020±0.0636 0.8544 0.031 0.0157±0.0898 0.5506 0.175 -5.7873±5.6998 +8.5±99.7 0.1196 0.085 0.5927

∆N3 0.0080±0.0595 0.4386 0.135 0.0267±0.0840 0.0727 0.318 -2.6899±3.1376 -47.1±56.0 **<0.0001** 0.841 0.1782

∆N3-REM* 0.0059±0.0268 0.8271 0.038 0.0103±0.0466 0.2069 0.221 3.0934±10.102 -55.9±177 0.0834 0.316 0.1885

**‘>median’ CBT-PBT (N=35, Fig. 3j):**

∆REM -0.0146±0.0641 0.1869 0.228 0.1466±0.0910 **<0.0001** 1.612 -3.1001±0.6216 -54.3±11.1 <**0.0001** 4.901 **<0.0001**

∆N3 0.0283±0.0577 **0.0056** 0.490 0.1006±0.0816 **<0.0001** 1.232 -0.4668±0.8149 -8.2±14.0 **0.0011** 0.586 **0.0001**

∆N3-REM* 0.0429±0.1184 **0.0382** 0.362 -0.0566±0.1026 **0.0020** 0.552 2.6333±0.1948 +46.1±3.5 **<0.0001** 13.18 **<0.0001**

**Legend to Suppl. Table 6.**

Results of the cosine fit analysis performed by the R -package ‘cosinoRmixedeffects’.

Mesor=7.5h mean value (min/10min); Amplitude= half of maximum-minimum value of the cosine fit (min/10min); Acro.=Acrophase= phase of maximum value of the cosine fit (2π*rad=360°=110min). rhythm. =rhythmicity p.

Median division of CBT-PBT gradient: ∆CBT-PBT values ≤median of ∆CBT-PBT/7.5h =’ ≤median’; ∆CBT-PBT values >median of ∆CBT-PBT/7.5h =’ >median’ (median ∆CBT-PBT= 0.2298°C/7.5h).

* indicates results derived from mixed effects models including random effects for mesor, amplitude and acrophase. The rhythm detection test (‘rhythmicity’), or zero-amplitude test, tests the overall significance of the cosinor model ^12^. d =effect size, Cohen’s d [small effect size (d) =0.2; medium d =0.5; large d =0.8; ^11^.

…….………….………….………….………….………….……….………………………………………………

Statistics for Figure 3

Figure 3b&e.

Mixed effects model-analyses revealed a significant time course of HM-LM for N3 (MATTRESS x TIME11, F(10,5470)=1.9267, p=0.0372) and REM (MATTRESS x TIME11, F(10,5470)=3.8793, p<0.0001).

Figure 3g&i.

Individuals with small nocturnal ∆CBT-PBT values (Fig. 3g, ≤ median) did not reveal a significant sleep-cycle pattern for ∆CBT-PBT [MATTRESS: F(1, 3024) = 55.003, p<0.0001; MATTRESS x TIME11: F(10, 3024) = 0.2390, p = 0.9924], and similarly, the ∆CBT-PBT sleep-cycle pattern of individuals with large nocturnal ∆CBT-PBT values (Fig. 3i, > median) is also not statistically significant [MATTRESS: F(1, 3024) = 1152.0, p<0.0001; MATTRESS x TIME11: F(10, 3024) = 0.1169, p = 0.9996] (Mixed effects model-analyses).

Figure 3h&j.

Mixed effects model-analyses of the time courses of individuals with small ∆CBT-PBT values (Fig.3h, ≤median) did not reach statistical significance in ∆N3 and ∆REM [N3, MATTRESS, F(1, 3024) = 0.4701, p=0.4926; MATTRESS x TIME11: F(10,3024=0.3944, p=0.9497; REM, MATTRESS: F(1, 3024) = 0.0342, p=0.8534; MATTRESS x TIME11: F(10,3024)=0.3272, p=0.9742]. The time courses of individuals with large ∆CBT-PBT values (Fig.3j, >median) showed a quit differentiated results for ∆N3 and ∆REM [N3, MATTRESS: F(1,3024)= 6.18552, p= 0.0129; MATTRESS x TIME11: N3, F(10,3024)=4.1840, p=0.0001; REM, MATTRTESS: F(1,3024)=1.6728, p=0.1960; F(10.3024)=8,7123, p<0.0001]. (Mixed effects model-analyses).

- - - - - - - -

**References**

1 Krauchi, K. & Deboer, T. The interrelationship between sleep regulation and thermoregulation. *Front Biosci (Landmark Ed)* **15**, 604-625 (2010).

2 Weinert, D. Circadian temperature variation and ageing. *Ageing Res Rev* **9**, 51-60 (2010).

3 Bliwise, D. L. Sleep in normal aging and dementia. *Sleep* **16**, 40-81 (1993).

4 Dijk, D. J., Duffy, J. F., Riel, E., Shanahan, T. L. & Czeisler, C. A. Ageing and the circadian and homeostatic regulation of human sleep during forced desynchrony of rest, melatonin and temperature rhythms. *J Physiol* **516 ( Pt 2)**, 611-627 (1999).

5 Monk, T. H., Buysse, D. J., Reynolds, C. F., 3rd, Kupfer, D. J. & Houck, P. R. Circadian temperature rhythms of older people. *Exp Gerontol* **30**, 455-474 (1995).

6 Vitiello, M. V. *et al.* Circadian temperature rhythms in young adult and aged men. *Neurobiol Aging* **7**, 97-100 (1986).

7 Zepelin, H. & McDonald, C. S. Age differences in autonomic variables during sleep. *J Gerontol* **42**, 142-146 (1987).

8 Huang, Y. L. *et al.* Age-associated difference in circadian sleep-wake and rest-activity rhythms. *Physiol Behav* **76**, 597-603 (2002).

9 Renfrew, J. W., Pettigrew, K. D. & Rapoport, S. I. Motor activity and sleep duration as a function of age in healthy men. *Physiol Behav* **41**, 627-634 (1987).

10 Blatteis, C. M. Age-dependent changes in temperature regulation - a mini review. *Gerontology* **58**, 289-295 (2012).

11 Cohen, J. *Statistical Power Analysis for the Behavioral Sciences*. 2 edn, (Routledge, 1988).

12 Cornelissen, G. Cosinor-based rhythmometry. *Theor Biol Med Model* **11**, 16 (2014).
